# Predicting the progression of chronic subdural hematoma based on skull density

**DOI:** 10.3389/fneur.2023.1279292

**Published:** 2023-10-20

**Authors:** Weijian Yang, Qifang Chen, Haijun Yao, Jun Zhang, Quan Zhang, Jiang Fang, Gang Wu, Jin Hu

**Affiliations:** ^1^Department of Neurosurgery, Huashan Hospital, Shanghai Medical College, Fudan University, Shanghai, China; ^2^National Center for Neurological Disorders, Shanghai, China; ^3^Shanghai Key Laboratory of Brain Function Restoration and Neural Regeneration, Shanghai, China; ^4^Neurosurgical Institute of Fudan University, Shanghai, China; ^5^Shanghai Clinical Medical Center of Neurosurgery, Shanghai, China; ^6^Department of Nursing, Huashan Hospital, Shanghai Medical College, Fudan University, Shanghai, China; ^7^Department of Neurosurgery and Neurocritical Care, Huashan Hospital, Shanghai Medical College, Fudan University, Shanghai, China

**Keywords:** chronic subdural hematoma, skull density, midline shift, surgery, progression

## Abstract

**Objective:**

The objective of this study was to investigate potential correlations between skull density and the progression of chronic subdural hematoma (CSDH).

**Methods:**

Patients with unilateral CSDH were retrospectively enrolled between January 2018 and December 2022. Demographic and clinical characteristics, as well as hematoma and skull density (Hounsfield unit, Hu), were collected and analyzed.

**Results:**

The study enrolled 830 patients with unilateral CSDH until the resolution of the CDSH or progressed with surgical treatment. Of the total, 488 patients (58.80%) necessitated surgical treatment. The study identified a significant correlation between the progression of CSDH and three variables: minimum skull density (MiSD), maximum skull density (MaSD), and skull density difference (SDD) (*p* < 0.001). Additionally, in the multivariable regression analysis, MiSD, MaSD, and SDD were independent predictors of CSDH progression. The MiSD + SDD model exhibited an accuracy of 0.88, as determined by the area under the receiver operating characteristic curve, with a sensitivity of 0.77 and specificity of 0.88. The model’s accuracy was validated through additional analysis.

**Conclusion:**

The findings suggest a significant correlation between skull density and the CSDH progression.

## 1. Introduction

Chronic subdural hematoma (CSDH), characterized by a collection of blood products in the subdural space, is likely to become the most common neurosurgical disease in adults by the year 2030 (1). The annual incidence of CSDH is approximately 13.3 per 100,000 patients in the general population, with significantly greater rates occurring in older adults at up to 58 per 100,000 (2). Surgical evacuation of the subdural hematoma is the main treatment modality for symptomatic patients (3). Research has indicated that the implementation of middle meningeal artery (MMA) embolization has yielded favorable outcomes in select individuals (4).

Anatomical considerations support the pathogenesis of CSDH and rationale for MMA embolization, as the blood supply to CSDH originates from MMA, which traverses the dura mater to establish connections with delicate vessels within the outer membrane of the hematoma (5). Recent research has revealed the existence of significant anastomosis among CSDH, dura, and skull (6). This suggests that there is a substantial interconnection between the blood supply of the skull and the dura mater, which may have a crucial role in the development of CSDH.

A significant correlation between the Hounsfield unit (Hu) values of the skull and bone mineral density has been previously established (7). Furthermore, it was reported that patients exhibiting lower bone mineral density are at a heightened risk of experiencing recurrent CSDH (8). As a result, we have formulated the hypothesis that skull density is correlated with the progression of CSDH.

## 2. Materials and methods

### 2.1. Participants and study settings

The studies involving humans were approved by the Ethics Committee of the Evaluation of Biomedical Research Projects of Huashan Hospital. The study was conducted in accordance with the local legislation and institutional requirements. Written informed consent for participation was not required from the participants or the participants’ legal guardians/next of kin in accordance with the national legislation and institutional requirements. The study evaluated all inpatient and outpatient individuals diagnosed with CSDH at Huashan Hospital, a university teaching hospital affiliated to Fudan University, from January 2018 to December 2022. The recruiting criteria were as follows: (1) patients presenting primary unilateral CSDH as diagnosed by computed tomography (CT) and (2) age ≥ 18 years. The exclusion criteria were as follows: (1) Patients with a history of tumor, intracerebral arteriovenous malformation, arachnoid cyst, ventriculoperitoneal shunts, immunodeficiency or thrombocytopenia, (2) Patients on immunosuppressants at admission, (3) Patients with bilateral or middle meningeal artery embolization, (4) Patients who were lost to follow-up. The patients were classified into two groups: recovery and progression. Recovery was defined as the resolution of CDSH on CT without surgical intervention. Hematoma progression was characterized by a significant decline in neurological function (such as worsening headache, progressive limb paralysis, or altered levels of consciousness) or by the observation of hematoma enlargement and/or midline shift (>1 cm) on follow-up CT scans (9). The patients were followed for a period of six months.

### 2.2. Data collection

**Figure 1:**
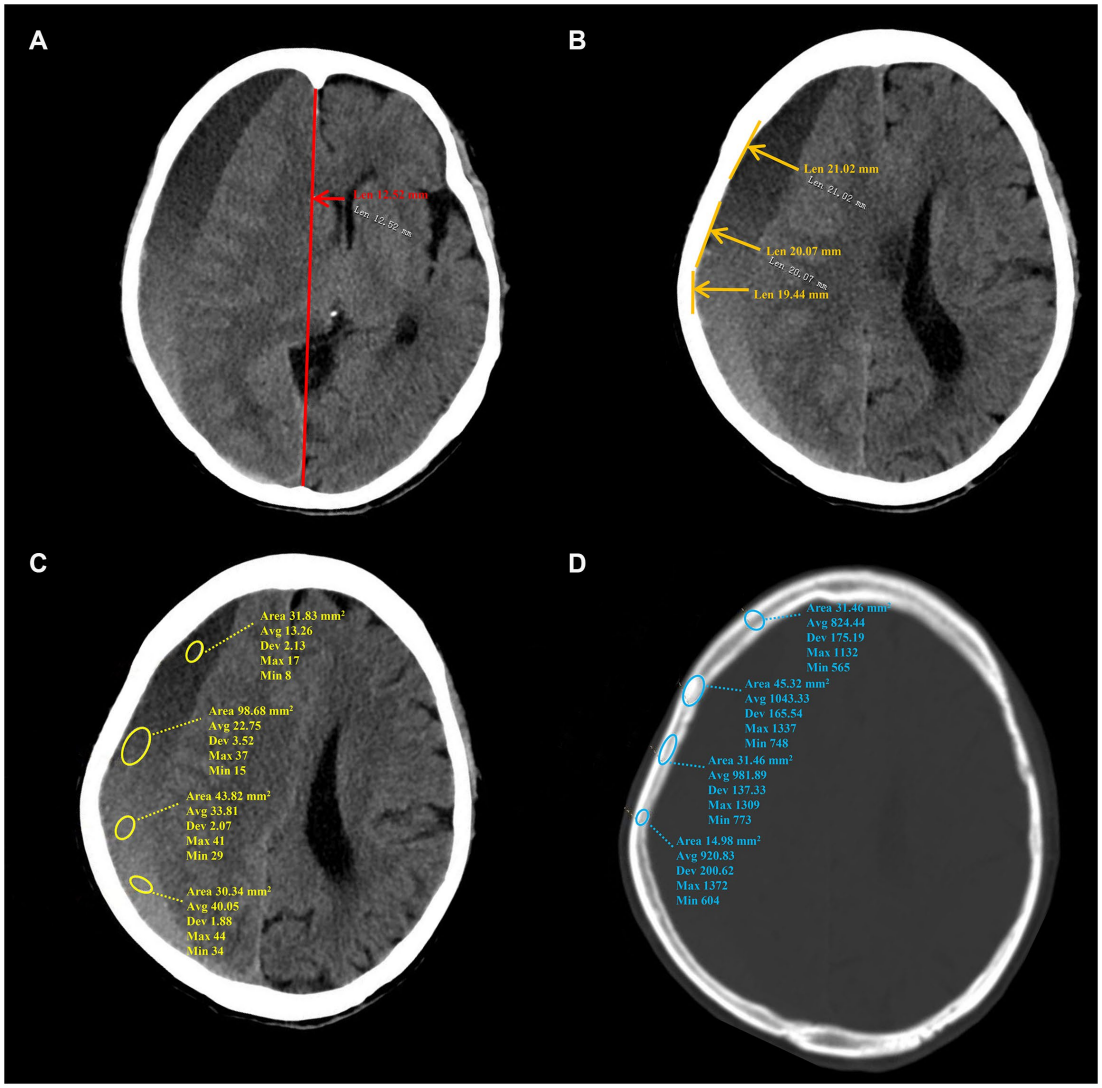
The data measurement method on CT. **(A–D)** Show the measurement method of MS, HT, hematoma density and skull density, separately. MS, midline shift; HT, maximal hematoma thickness.

The study collected demographic information, clinical characteristics, hematoma density, and skull density of enrolled patients. The collected data encompassed gender, age, etiology, clinical symptoms, medical history, therapeutic records, and CT data. The following CT data were acquired from the initial diagnosis: initial midline shift (IMS) and initial hematoma thickness (IHT). The following CT data were acquired at the time of peak midline shift (MS) and/or maximal hematoma thickness (HT). The CT data comprised MS, HT in the axial plane, minimum hematoma density (MiHD), maximal hematoma density (MaHD), minimum skull density (MiSD), and maximal skull density (MaSD). The present study employed millimeters (mm) as the unit of measurement for both MS and HT. Hu was utilized to determine the density value for CT data. The MS was operationally defined as the line extending from the septum pellucidum, perpendicular to the midline, between the most anterior and posterior parts of the falx cerebri (10). HT was measured from the brain surface, perpendicular to the tangent line of inner compact bone. Hematoma density difference (HDD) was computed as the difference between the MaHD and the MiHD. Skull density was measured at the level of maximum hematoma thickness. Skull density difference (SDD) was calculated as the difference between MaSD and the MiSD. The data measurement method on CT is depicted in Figure 1. To ensure accuracy, three neurosurgeons from the university faculty confirmed all radiologic findings. The clinical data was blinded using the picture archiving and communication system (PACS).

### 2.3. Statistical analysis

Continuous data with a normal distribution were expressed as mean ± standard deviation (–X ± SD). The student’s unpaired t-test or Chi-square test was used to compare variables between the groups. Enumeration data were described in frequency or percentage. Univariable and multivariable logistic regression models were employed to assess the patient parameters. Receiver operating characteristic (ROC) curve analysis was subsequently adopted to discriminate the capacity of this model. The Youden index was used to identify sensitivity and specificity based on the coordinate points of the highest combination of these two parameters on ROC curve. The model’s capacity for discrimination, accuracy in prediction, and clinical utility were evaluated through the utilization of a ROC curve, calibration plot, and decision curve analysis. To mitigate overfit bias, one thousand bootstrap resamples were utilized. SPSS 20.0 software (The IBM SPSS software Company) was used for statistical analysis, and *p* < 0.05 were considered statistically significant. The visualization figures were produced by Darwin^1^ (accessed on October 4, 2023) and genescloud^2^ (accessed on October 4, 2023), while the pattern diagram was generated by Figdraw^3^ (accessed on October 4, 2023).

## 3. Results

### 3.1. Demographic and clinical characteristics of patients

For statistical analysis, a cohort of 830 patients diagnosed with unilateral CSDH was included. The mean age of the cohort was 68.53 ± 12.99 years, with 669 (80.60%) males. The incidence of CSDH was highest among patients aged 61 to 80 years, accounting for 65.18% of cases. The most commonly observed clinical symptom was headache (45.33%), followed by dyskinesia (37.63%) and impaired awareness (5.71%). The relevant data is presented in Table 1.

**Table 1:** Demographic and clinical characteristics of patients.

Variable	*N* = 830
Sex (male)	669 (80.60%)
Age (years, mean ± SD)	68.53 ± 12.99
<60	166 (20.00%)
61 ≤ Age < 80	541 (65.18%)
≥81	123 (14.82%)
Neurological deficits	
Headache	524 (45.33%)
Dyskinesia	435 (37.63%)
Impaired awareness	66 (5.71%)
Aphasia	61 (5.28%)
Amnesia	51 (4.41%)
Seizure	19 (1.64%)
History of head trauma	
Yes	514 (61.93%)
No	316 (38.07%)
Antithrombotic drug	
Yes	89 (10.72%)
No	741 (89.28%)
Group	
Recovery group	342 (41.20%)
Progression group	488 (58.80%)

Among the cohort of 830 patients, a majority of 514 individuals (61.93%) reported experiencing head trauma within the preceding three months, while 316 (38.07%) did not report any such incidents. Of the total, 89 patients (10.72%) were undergoing long-term antithrombotic treatment at the time of CSDH confirmation through neuroimaging, while the remaining 741 (89.28%) were not receiving such therapy. The patients were subsequently categorized into two groups based on their recovery status, with 342 (41.20%) individuals classified as belonging to the recovery group and 488 (58.80%) to the progression group. The relevant data is presented in Table 1.

### 3.2. Varied characteristics of recovery and progression patients

The analysis revealed no statistically significant differences in sex, anticoagulation therapy and antiplatelet therapy between the two groups, with *p* values of 0.09, 0.98 and 0.50, respectively. However, age, history of head trauma, and all CT characteristics exhibited significant differences between the recovery and progression patients, with a *p* value of less than 0.001. The detailed data can be found in Table 2.

**Table 2:** Varied characteristics of patients in recovery and progression group.

Variables	Recovery group	Progression group	*P*
	*N* = 342 (41.20%)	*N* = 488 (58.80%)	
Sex (male)			0.09
Female	76 (22.22%)	85 (17.42%)	
Male	266 (77.78%)	403 (82.58%)	
Age (years, mean ± SD)	66.86 ± 14.35	69.70 ± 11.83	0.002
<60	87 (25.44%)	79 (16.19%)	
61 ≤ Age < 80	205 (59.94%)	336 (68.85%)	
≥81	50 (14.62%)	73 (14.96%)	
History of head trauma			
Yes	240 (70.18%)	274 (56.15%)	< 0.001
No	102 (29.82%)	214 (43.85%)	
Anticoagulation therapy			0.98
Yes	9 (2.63%)	13 (2.66%)	
No	333 (97.37%)	475 (97.34%)	
Antiplatelet therapy			0.50
Yes	25 (7.31%)	42 (8.61%)	
No	317 (92.69%)	446 (91.39%)	
CT characteristics			
IMS (mm, mean ± SD)	3.74 ± 2.16	3.98 ± 2.86	0.17
IHT (mm, mean ± SD)	9.45 ± 5.42	9.57 ± 4.91	0.74
MS (mm, mean ± SD)	5.70 ± 2.20	12.63 ± 2.94	< 0.001
HT (mm, mean ± SD)	18.89 ± 6.34	26.28 ± 5.40	< 0.001
MiHD (Hu, mean ± SD)	22.43 ± 8.88	25.24 ± 10.43	< 0.001
MaHD (Hu, mean ± SD)	40.04 ± 10.29	52.77 ± 9.28	< 0.001
HDD (Hu, mean ± SD)	17.61 ± 8.98	27.53 ± 10.21	< 0.001
MiSD (Hu, mean ± SD)	701.70 ± 118.17	565.09 ± 151.37	< 0.001
MaSD (Hu, mean ± SD)	1378.63 ± 157.68	1500.60 ± 193.99	< 0.001
SDD (Hu, mean ± SD)	676.93 ± 126.66	935.51 ± 204.10	< 0.001

IMS, Initial Midline shift; IHT, Initial Hematoma thickness; MS, Midline shift; HT, Hematoma thickness; MiHD, Min Hematoma density; MaHD, Max Hematoma density; HDD, Hematoma density difference; MiSD, Min skull density; MaSD, Max skull density; SDD, Skull density difference.

**Figure 2:**
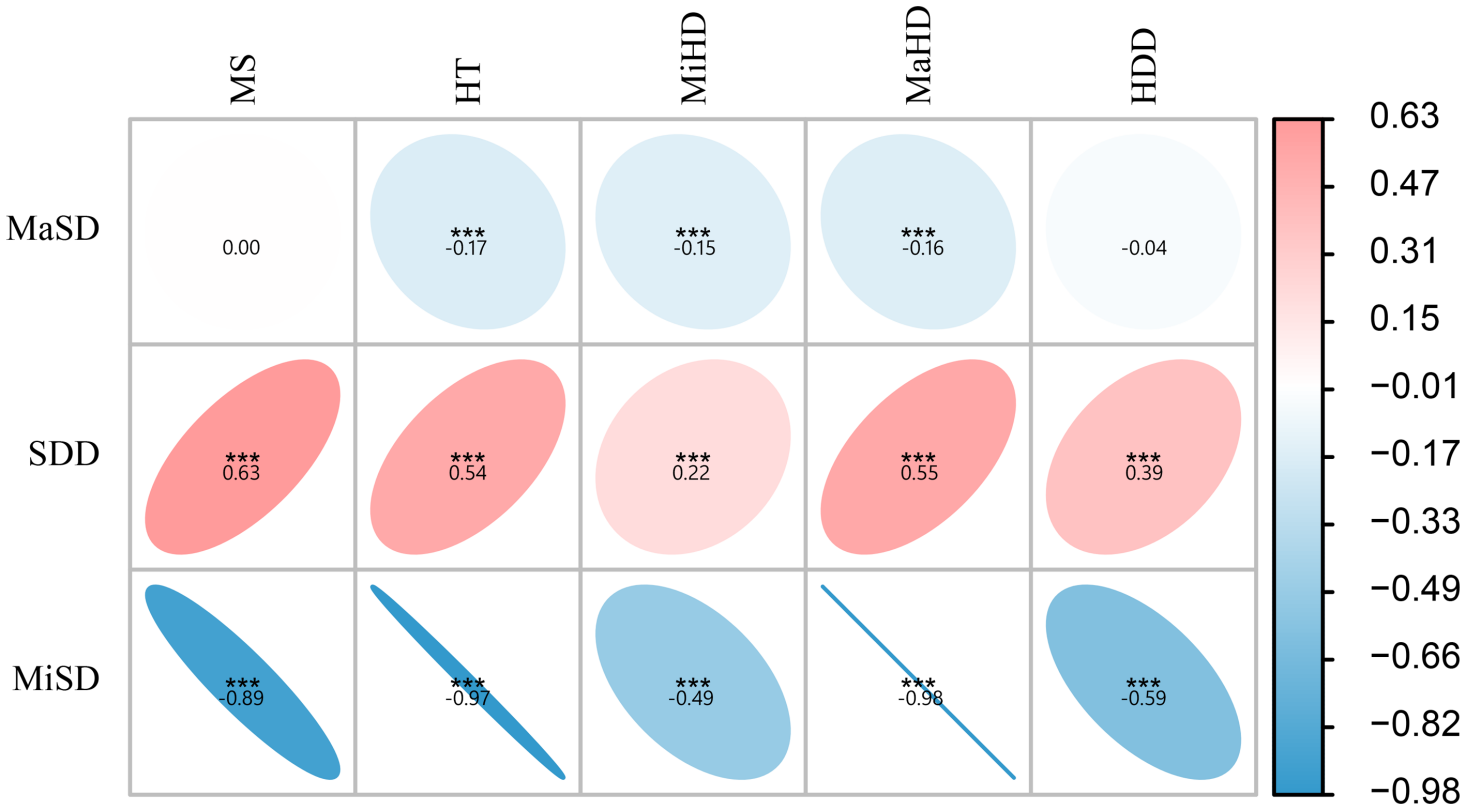
Correlation analysis between skull density and hematoma density. ***p < 0.001, otherwise *p* > 0.05. The data in the figure represent the correlation coefficients. The elliptical shape appears more elongated (approaching a straight line), suggesting a linear relationship.

The present study conducted a further analysis of the relationship between skull density parameters (MiSD, MaSD, SDD) and CT data of hematoma (MS, HT, MiHD, MaHD, HDD). The results indicated a positive correlation between SDD and CT data of hematoma (MS, HT, MiHD, MaHD, HDD), albeit with a small correlation coefficient. Specifically, the correlation coefficient between SDD and MS was found to be the highest (0.63). In contrast, MiSD displayed a strong inverse correlation with CT data of hematoma (MS, HT, MiHD, MaHD, HDD), exhibiting correlation coefficients of −0.89, −0.97 and −0.98, respectively. The elliptical shape appears more elongated (approaching a straight line), suggesting a linear relationship. The results suggest a linear correlation between MiSD and hematoma CT data. These findings are presented in Figure 2.

### 3.3. Skull density related to CSDH progression

Following the univariable and Correlation analyses, a multivariable logistic regression analysis was conducted to examine the variables that displayed statistical significance. The results indicated that MiSD, MaSD, and SDD were significant independent predictors of CSDH progression in the multivariable model (*p* < 0.001), as presented in Table 3.

**Table 3:** Multivariable logistic regression analysis of predictors of CSDH progression.

Factor	*B*	SE	Wald	df	Sig.	Exp (B) (95% CI)
MiSD	−0.0039	0.0007	30.8587	1	< 0.001	0.996 (0.995–0.997)
MaSD	0.0080	0.001	142.7102	1	< 0.001	1.008 (1.007–1.009)
SDD	0.0091	0.001	190.6521	1	< 0.001	1.009 (1.008–1.010)

B, regression coefficient; SE, standard error; Wald, Wald score; df, degrees of freedom; Sig., level of significance; MiHD, Min Hematoma density; MiSD, Min skull density; MaSD, Max skull density; SDD, Skull density difference.

**Figure 3:**
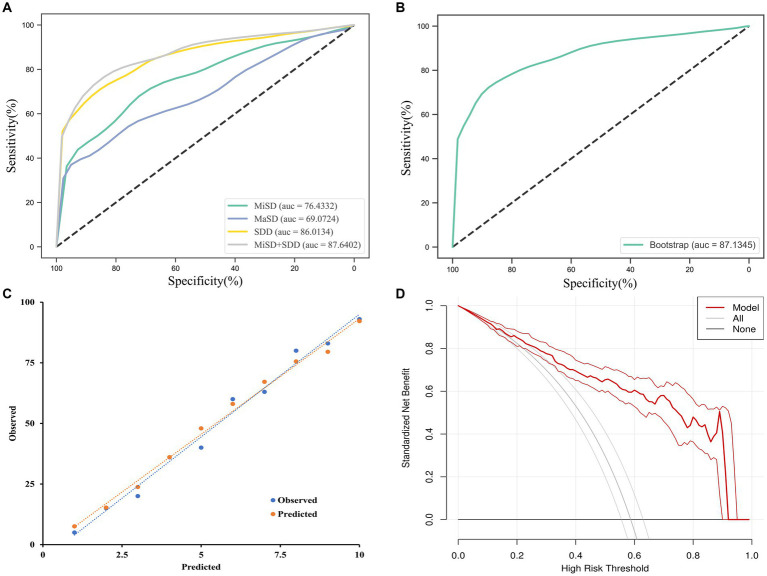
ROC curves analysis and evaluation of the model. **(A)** The ROC curves for the MiSD, MaSD, SDD and MiSD+SDD. **(B)** The ROC curve for the MiSD+SDD model generated using bootstrap resampling (1,000 times). **(C)** Calibration plot. The closer the blue calibration curve and the red standard curve are, the better the calibration ability of the model is suggested. **(D)** Decision curve analysis for the MiSD+SDD model. The red line is from the MiSD+SDD model, the gray line indicates progression, and the horizontal line indicates recovery. The graph depicts the expected standardized net benefit per patient relative to the prediction of progression. The standardized net benefit increases as the model curve is extended. MiHD, Min Hematoma density; MiSD, Min skull density; MaSD, Max skull density; SDD, Skull density difference.

The independent predictors derived from the multivariate logistic regression model were utilized in constructing the ROC model. While the multivariate analysis did not demonstrate a significant enhancement in efficacy, it did exhibit a potential for improving sensitivity as illustrated in Figure 3A and Table 4. The variables’ cut-off, sensitivity, specificity, Youden index, and AUC values are presented in Table 4.

**Table 4:** Prognostic accuracy of CSDH progression.

Variables	Cut-off	Sensitivity	Specificity	Youden index	AUC
MiSD (Hu)	634	0.73	0.71	0.44	0.76
MaSD (Hu)	1,597	0.39	0.96	0.35	0.69
SDD (Hu)	830	0.69	0.89	0.58	0.86
MiSD + SDD	-	0.77	0.88	0.65	0.88

AUC, area under the curve; GCS, Glasgow Coma Scale; MiHD, Min Hematoma density; MiSD, Min skull density; MaSD, Max skull density; SDD, Skull density difference.

### 3.4. Internal validation, calibration, and decision curve analysis

The accuracy of the MiSD+SDD model was confirmed through a bootstrap method involving 1,000 resamples, resulting in an AUC of 0.87 (Figure 3B). Furthermore, the predicted probabilities generated by the MiSD+SDD model exhibited a strong correlation with the clinical outcomes (Figure 3C), and the decision curve analysis demonstrated the potential clinical utility of the model (Figure 3D).

## 4. Discussion

This is the first study to investigate the association between skull density and CSDH progression. Our findings indicate that MiSD, MaSD, and SDD are all independent risk factors for the progression of CSDH. Furthermore, MiSD and SDD were utilized to construct a model for the assessment and validation of CSDH progression, which is a crucial step in identifying this condition.

With the development of population aging, the prevalence of CSDH is expected to escalate annually, ultimately emerging as a prominent disease posing a significant threat to the elderly population (11). The preferred method of treatment for CSDH is surgical evacuation of the hematoma via Burr-hole with drainage (12). Despite the widespread acceptance of the aforementioned surgical approach, several alternative options including bedside twist drill craniotomy with placement of a subdural drain or subdural evacuation port system, endoscope-assisted evacuation and MMA embolization have been increasingly implemented. Bedside subdural evacuation port system placement has gained popularity due to its ability to eliminate the requirement for general anesthesia and the need for availability of an operating room. Consequently, patients with multiple comorbidities are more inclined to undergo this procedure (13). The utilization of endoscope-assisted evacuation in conjunction with MMA embolization has the potential to effectively reduce the recurrence of CSDH by improving hematoma evacuation, removing membranes, coagulating at-risk blood vessels, strategically placing surgical drains, assessing embolization efficacy, coagulating fragile membranes, and eliminating acute clot (14). The formation of subdural hematoma involves the MMA and its branches, and MMA embolization has been found to have a therapeutic effect (15). Despite surgery being the primary treatment for CSDH, the recurrence rate post-surgery varies significantly, and drug therapy is also a viable treatment option for CSDH (16). Nonetheless, the absence of concrete evidence-based guidelines for surgical indications (17), coupled with divergent reports that suggest a case-by-case approach to surgery (18, 19).

**Figure 4:**
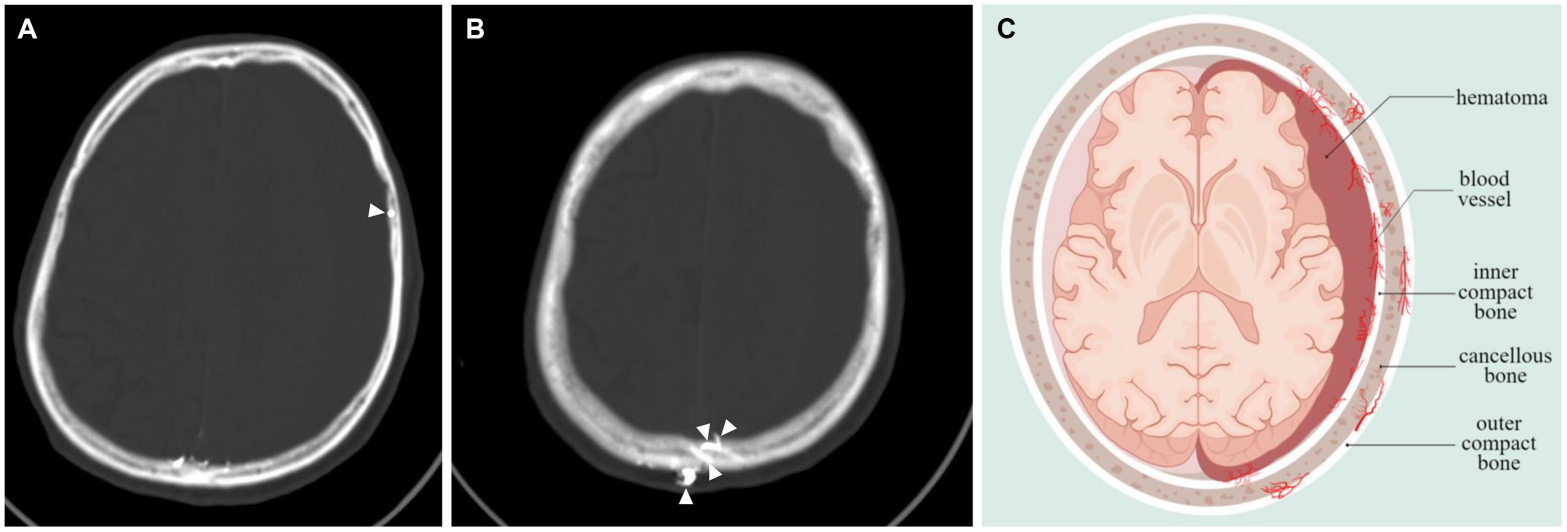
Vascular anastomosis of hematoma, dura and skull. **(A,B)** Middle meningeal artery embolization revealed extensive anastomoses vessels between dura and skull (indicated by arrows). **(C)** Hematoma-dura-skull relationships, emphasizing the “vascular anastomosis” principle.

Underscores the need to determine the CSDH progression, as this will significantly influence the available treatment options. Our study showed that antithrombotic therapy is not associated with CSDH volume progression, which is consistent with the results of others (20). Although there were statistically significant differences in age and of trauma between the two groups, we focused on the correlation between skull density and the progression of hematoma. It was found that the vessels of the dura exhibited significant anastomoses with the vessels of the skull (6). Furthermore, postoperative recurrence was found to be associated with skull density (8). Empirical evidence has demonstrated that patients with MMA embolization display extensive anastomosis among subdural hematoma, dura, and skull, as depicted in Figures 4A,B. The pattern diagram in Figure 4C provides a detailed illustration of the anastomosis among hematoma, dura, and skull. Consequently, it is hypothesized that skull density is linked to the progression of CSDH.

Various radiological measures, including the midline shift, brain atrophy, hematoma density, and maximum width of the hematoma, are utilized to assess the extent and space-occupying effect of different intracranial masses (10, 11). Previous studies have shown a significant correlation between Hu values and bone mineral density (21). Furthermore, it was reported that patients with lower bone mineral density were at a higher risk of CSDH recurrence (8). The present study reveals that patients with CSDH progression exhibit lower MiSD, higher MaSD and SDD. The findings indicate a negative correlation between MiSD and hematoma progression. Furthermore, in the multi-variable regression analysis, MiSD, MaSD, and SDD emerged as significant independent predictors of CSDH progression. The accuracy of the MiSD+SDD model, as estimated by the area under the ROC, was 0.88 (with a sensitivity of 0.77 and specificity of 0.88). The validation analysis confirmed the high accuracy of the model.

The treatment options for CSDH display variations in terms of indication, timing, and type of surgical intervention, duration of drainage, concomitant membranectomy, and the necessity of MMA embolization (22). We found that patients with CSDH progression exhibit lower MiSD (Cut-off 634 Hu), higher MaSD (Cut-off 1,597 Hu) and SDD (Cut-off 830 Hu). The findings of this study may have significant implications for the early assessment of CSDH progression and the determination of the necessity for surgical intervention. The use of MMA embolization shows promise in preventing re-bleeding and recurrence (23). The meningeal branch of the MMA is highly extensive, encompassing various regions such as the orbital, frontal, top, occipital, posterior fossa, and base of the middle cranial fossa, depending on the blood supply location (24). The literature states that the meningeal arteries provide blood supply to both the skull and dura mater (25). A study has indicated that the distal penetration of the liquid embolic agent may be linked to a faster clearance of hematoma (26). The findings imply that the branch of the MMA supplying the skull was subjected to embolization. Our investigation revealed a correlation between skull density and the progression of CSDH. This finding holds significant value in terms of facilitating the early detection of hematoma progression and the timely implementation of MMA embolization as a treatment approach. However, additional research is required to ascertain the potential correlation between skull density and the effectiveness of MMA embolization in mitigating the recurrence of CSDH.

Nonetheless, it is crucial to note that the interpretation of these findings is subject to three significant caveats. Firstly, as with prior retrospective case–control studies, the ability to establish causal inference is constrained. Secondly, despite the confirmation of all radiologic findings by three faculty neurosurgeons who were blinded to the clinical data using PACS, the measurement may have been influenced by individualistic differences, leading to potential selection bias. Lastly, while our model was validated using bootstraps with 1,000 resamples, further prospective multicenter studies are necessary to externally validate our results.

## 5. Conclusion

The findings of the study indicate that lower MiSD, higher MaSD and higher SDD are significantly correlated with CSDH progression. To enhance the predictive accuracy of CSDH progression and facilitate treatment decision-making, we constructed a MiSD+SDD model and validated it through 1,000 bootstrap resamples. This model holds promise for clinical application.

## Data availability statement

The original contributions presented in the study are included in the article/supplementary material, further inquiries can be directed to the corresponding authors.

## Ethics statement

The studies involving humans were approved by the Ethics Committee of the Evaluation of Biomedical Research Projects of Huashan Hospital. The studies were conducted in accordance with the local legislation and institutional requirements. Written informed consent for participation was not required from the participants or the participants’ legal guardians/next of kin in accordance with the national legislation and institutional requirements.

## Author contributions

WY: Data curation, Funding acquisition, Writing – original draft. QC: Data curation, Writing – review & editing. HY: Data curation, Writing – review & editing. JZ: Formal analysis, Writing – review & editing. QZ: Formal analysis, Writing – review & editing. JF: Formal analysis, Writing – review & editing. GW: Conceptualization, Investigation, Methodology, Project administration, Resources, Supervision, Validation, Writing – review & editing. JH: Conceptualization, Funding acquisition, Methodology, Project administration, Resources, Supervision, Validation, Writing – review & editing.

## Footnotes

^1^https://darwin-online.yizhun-ai.com/

^2^https://www.genescloud.cn

^3^https://www.figdraw.com/static/index.html
